# 

**Published:** 1915-02-27

**Authors:** 


### CONTENTS.

PAGE

**Military Medical Heroism** ... ... ... ... ... 477

**Open Posts and Poor-Law Volunteers** ... ... 478

**Hospital and Institutional News** ... ... ... 479

Honours for Army Surgeons—The Double V.C. : Unprecedented Honour for a Surgeon—The Retention of Post-mortem Specimens : The Leeds Infirmary Case—The Government and Infirmary Coal Supplies—The Death of a Great Matron—Florence Nightingale's Statue Unveiled—The Nightingale Annuities Scheme—The First London County Councillor to be Wounded—Casualties in the German Medical Service—A Staff Scarcity at Worcester Infirmary—The Birmingham System of Voluntary Aid—Dr. E. C. Austin's New Post—North Evington Infirmary as a Supplemental Base Hospital?—Advantages of such a Scheme—The Rearrangement of Local Administration—Mental Hospital Laundry Work—The Education of the Hospital Officer—The Present Discontent—Fifty Years as Secretary—A Hospital in the Antarctic—The Saturday Fund in War Time—A Pioneer Medical Superintendent—St. Mark's as a Cancer Hospital—Should the Poor Law Subsidise Sanatoria?—The Epsom Hospital Affair—The "Blockade" and the Drug Market.

PAGE

**War and the Anti-Tuberculosis Crusade** ... 485

The Effect of Military Enlistment.

**Gunshot Wounds of the Head** ... ... ... 486

The Collective Experience of Surgeons.

**Hygienic Conditions at the Crystal Palace** ... 487

Have Sufficient Precautions been Taken?

**The School Doctor and Slight Ailments** ... 489

Chronic Catarrhal Appendicitis in School Children.

[*See over.*]

### CONTENTS—(*continued*).

PAGE

**Round the World** ... ... ... ... ... ... 488

Fellow-Workers and the Roll of Honour.

**The London Panel Committee Meeting** ... 490

Conditions of Service: The New System of Certification.

**The New Soldiers' Wards at Norwich Hospital** ... ... ... ... ... ... 491

A Suggestive Type for Continued Treatment.

**Awards to the Medical Services** ... ... ... 492

**The Editor's Letter-Box** ... ... ... ... 493

The Education of Hospital Officers.

Hospital Administrators at the Base Hospitals in South Africa.

The Clinical Value of Poor-Law Infirmaries.

**The late Founder of "King's" Training School** ... ... ... ... ... ... 493

PAGE

**A Gravesend Good Samaritan** ... ... ... 494

**Gifts and Bequests** ... ... ... ... ... ... 494

**The Book Market** ... ... ... ... ... ... 494

**The Department of Modern Nursing** ... ... 495

IX.—Guy's Hospital.

**From the Patient's Point of View** ... ... 498

Three Weeks at a Seaside Convalescent Home.

**The Institutional Worker** ... ... (Suppl.) 1

Answer to January Question: Receiving Goods Without a Steward.

Institutional Workers and their Work.

Question for February.

Questions Invited.

Employment and Training.

The Sick and in Need.

